# A butterfly-shaped acceptor with rigid skeleton and unique assembly enables both efficient organic photovoltaics and high-speed organic photodetectors

**DOI:** 10.1093/nsr/nwae409

**Published:** 2024-11-13

**Authors:** Wendi Shi, Qiansai Han, Yu Zhu, Yingjun Xia, Tengfei He, Shijie Wang, Longyu Li, Wei Ma, Guankui Long, Guanghui Li, Zhaoyang Yao, Chenxi Li, Xiangjian Wan, Yongsheng Chen

**Affiliations:** State Key Laboratory of Elemento-Organic Chemistry, Frontiers Science Center for New Organic Matter, The Centre of Nanoscale Science and Technology and Key Laboratory of Functional Polymer Materials, Institute of Polymer Chemistry, Renewable Energy Conversion and Storage Center (RECAST), Tianjin Key Laboratory of Functional Polymer Materials, Nankai University, Tianjin 300071, China; State Key Laboratory of Elemento-Organic Chemistry, Frontiers Science Center for New Organic Matter, The Centre of Nanoscale Science and Technology and Key Laboratory of Functional Polymer Materials, Institute of Polymer Chemistry, Renewable Energy Conversion and Storage Center (RECAST), Tianjin Key Laboratory of Functional Polymer Materials, Nankai University, Tianjin 300071, China; State Key Laboratory of Elemento-Organic Chemistry, Frontiers Science Center for New Organic Matter, The Centre of Nanoscale Science and Technology and Key Laboratory of Functional Polymer Materials, Institute of Polymer Chemistry, Renewable Energy Conversion and Storage Center (RECAST), Tianjin Key Laboratory of Functional Polymer Materials, Nankai University, Tianjin 300071, China; State Key Laboratory of Elemento-Organic Chemistry, Frontiers Science Center for New Organic Matter, The Centre of Nanoscale Science and Technology and Key Laboratory of Functional Polymer Materials, Institute of Polymer Chemistry, Renewable Energy Conversion and Storage Center (RECAST), Tianjin Key Laboratory of Functional Polymer Materials, Nankai University, Tianjin 300071, China; State Key Laboratory of Elemento-Organic Chemistry, Frontiers Science Center for New Organic Matter, The Centre of Nanoscale Science and Technology and Key Laboratory of Functional Polymer Materials, Institute of Polymer Chemistry, Renewable Energy Conversion and Storage Center (RECAST), Tianjin Key Laboratory of Functional Polymer Materials, Nankai University, Tianjin 300071, China; State Key Laboratory for Mechanical Behavior of Materials, Xi'an Jiaotong University, Xi'an 710049, China; State Key Laboratory of Elemento-Organic Chemistry, Frontiers Science Center for New Organic Matter, The Centre of Nanoscale Science and Technology and Key Laboratory of Functional Polymer Materials, Institute of Polymer Chemistry, Renewable Energy Conversion and Storage Center (RECAST), Tianjin Key Laboratory of Functional Polymer Materials, Nankai University, Tianjin 300071, China; State Key Laboratory for Mechanical Behavior of Materials, Xi'an Jiaotong University, Xi'an 710049, China; School of Materials Science and Engineering, National Institute for Advanced Materials, Renewable Energy Conversion and Storage Center (RECAST), Nankai University, Tianjin 300350, China; State Key Laboratory of Elemento-Organic Chemistry, Frontiers Science Center for New Organic Matter, The Centre of Nanoscale Science and Technology and Key Laboratory of Functional Polymer Materials, Institute of Polymer Chemistry, Renewable Energy Conversion and Storage Center (RECAST), Tianjin Key Laboratory of Functional Polymer Materials, Nankai University, Tianjin 300071, China; State Key Laboratory of Elemento-Organic Chemistry, Frontiers Science Center for New Organic Matter, The Centre of Nanoscale Science and Technology and Key Laboratory of Functional Polymer Materials, Institute of Polymer Chemistry, Renewable Energy Conversion and Storage Center (RECAST), Tianjin Key Laboratory of Functional Polymer Materials, Nankai University, Tianjin 300071, China; State Key Laboratory of Elemento-Organic Chemistry, Frontiers Science Center for New Organic Matter, The Centre of Nanoscale Science and Technology and Key Laboratory of Functional Polymer Materials, Institute of Polymer Chemistry, Renewable Energy Conversion and Storage Center (RECAST), Tianjin Key Laboratory of Functional Polymer Materials, Nankai University, Tianjin 300071, China; State Key Laboratory of Elemento-Organic Chemistry, Frontiers Science Center for New Organic Matter, The Centre of Nanoscale Science and Technology and Key Laboratory of Functional Polymer Materials, Institute of Polymer Chemistry, Renewable Energy Conversion and Storage Center (RECAST), Tianjin Key Laboratory of Functional Polymer Materials, Nankai University, Tianjin 300071, China; State Key Laboratory of Elemento-Organic Chemistry, Frontiers Science Center for New Organic Matter, The Centre of Nanoscale Science and Technology and Key Laboratory of Functional Polymer Materials, Institute of Polymer Chemistry, Renewable Energy Conversion and Storage Center (RECAST), Tianjin Key Laboratory of Functional Polymer Materials, Nankai University, Tianjin 300071, China

**Keywords:** organic photovoltaics, organic photodetectors, electron acceptor, efficiency, response speed

## Abstract

It remains challenging to design efficient bifunctional semiconductor materials in organic photovoltaic and photodetector devices. Here, we report a butterfly-shaped molecule, named WD-6, which exhibits low energy disorder and small reorganization energy due to its enhanced molecular rigidity and unique assembly with strong intermolecular interaction. The binary photovoltaic device based on PM6:WD-6 achieved an efficiency of 18.41%. Notably, an efficiency of 19.42% was achieved for the ternary device based on PM6:BTP-eC9:WD-6. Moreover, the photodetection device based on WD-6 demonstrated an ultrafast response speed (205 ns response time at λ of 820 nm) and a high cutoff frequency of −3 dB (2.45 MHz), surpassing the values of most commercial Si photodiodes. Based on these findings, we showcased an application of the WD-6-based photodetection device in high-speed optical communication. These results offer valuable insights into the design of organic semiconductor materials capable of simultaneously exhibiting high photovoltaic and photodetective performance.

## Teaser

A butterfly-shaped bifunctional acceptor named WD-6 has been designed, which achieves a high efficiency of 19.42% in organic photovoltaics and an ultrafast response speed of 205 ns in organic photodetectors.

## INTRODUCTION

As technology evolves, particularly wearables, soft robotics, the Internet of Things and other emerging technologies, the demand for sustainable energy and high-speed data transmission continues to increase [1–3]. One potential approach to addressing this challenge is the development of flexible and lightweight devices that simultaneously provide renewable energy and transmit data at high speeds. Organic optoelectronic devices, such as organic photovoltaics (OPVs) [4] and organic photodetectors (OPDs) [1] based on organic semiconductors with π-conjugated molecular backbones, have made significant strides in promising applications for renewable energy sources and information reception. Moreover, wireless optical communication systems utilizing high-speed OPDs are considered one of the most compelling wireless communication technologies for high-speed and large-capacity data communication [5]. In fact, state-of-the-art OPDs based on the diode structure and bulk heterojunction active layer share very similar material systems with OPVs. However, it is worth noting that organic semiconductor materials with good photovoltaic performance may not necessarily show good photodetective properties. Thus, designing organic semiconductor materials with high performance in both OPVs and OPDs remains challenging.

Compared to inorganic semiconductors with highly ordered structures, organic semiconductor materials, particularly those processed in solution for device fabrication, commonly exhibit more structural defects and amorphous phases, resulting in large trap densities [6,7]. In the case of OPVs, trap sites in photoactive films serve as trap-assisted recombination centers, contributing to non-radiation recombination under illumination, thus reducing the short circuit current density (*J_SC_*) and decreasing the maximum achievable open circuit voltage (*V*_OC_) [8]. In OPD devices, trap sites narrow the thermal barriers for charges in the dark, thus leading to a high dark current and noise current [9,10]. Moreover, thermally excited charges can jump from the highest occupied molecular orbital (HOMO) of donors to the lowest unoccupied molecular orbital (LUMO) of acceptors via the trapping and de-trapping process with the assistance of trap states, which further improves the noise current and limits charge mobilities of organic photoactive films [11,12]. Consequently, OPDs exhibit low sensitivity and slow response speed, hindering their applications in optical communication. Therefore, it is crucial to minimize the trap state to fabricate high-efficiency OPVs and high-speed OPDs for renewable energy and high-speed data communication.

Although the trap state is determined by many complicated factors in bulk heterojunction (BHJ) films, the chemical structures and molecular configurations of active layer materials always play a fundamental role. Several studies have been reported to reduce the trap density of photovoltaic active layer materials by designing and optimizing the molecular configuration through extending π-conjugation. For example, Baran *et al.* demonstrated that extending π-conjugation along with molecular backbones increased the molecular packing, coherence length and domain purity, and thus decreased the trap-assisted charge recombination [13]. Zhao *et al.* also found that the π-conjugation of the donor core in non-fullerene acceptors from heptacyclic units to nonacyclic units could efficiently reduce the trap density in OPVs [14]. Compared to 1D conjugation, 2D conjugation expansion can strengthen intermolecular interactions and improve the electronic coupling and charge transfer ability, thus providing more opportunities to reduce trap densities [15–18]. Recently, dimers and trimers based on the Y6 skeleton have been extensively studied and have shown promising photovoltaic performance [17,19–22]. However, most of them are linked by small aromatic units or alkyl chains and cannot form planar and rigid conjugations [23,24]. In addition, few studies focused on the correlation between the molecular structure, trap density and device performance.

**Figure 1.**
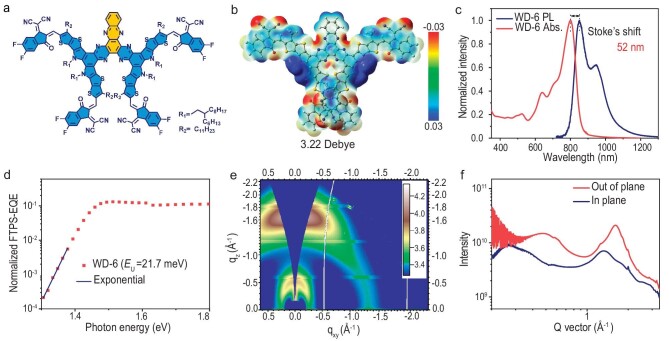
(a) Chemical structures of WD-6. (b) The surface electrostatic potential contour of WD-6. (c) UV–vis absorption (abs.) and photoluminescence (PL) spectra of acceptors in the films. (d) FTPS-EQE of the WD-6-based devices at the absorption onset. (e) 2D GIWAXS patterns of WD-6-based neat film. (f) Line-cut profiles of WD-6 neat film.

Recently, our group reported a series of central core conjugation extension acceptors (CH-series) [18]. Owing to the 2D conjugation extension, those acceptors show multimodal and strong 3D intermolecular packing, leading to reduced energy disorder and small reorganized energy compared with Y6. In this study, based on the CH molecule design rationale, we have designed and synthesized a molecule, named WD-6 (Fig. 1a), which is characterized by a 2D extended conjugation and rigid molecular backbone. WD-6 demonstrates low energy disorder with impressive low Urbach energy (*E*_U_) (21.7 meV) and small reorganization energy (λ_electron_ = 96.38 meV, λ_hole_ = 108.44 meV), indicating efficient intermolecular interactions in the neat film. Notably, the PM6:WD-6 blend film shows a trap density of 6.77 × 10^15^ cm^−3^, lower than that of organic semiconductors in OPVs with a value of 10^16^–10^18^ cm^−3^ [25], which facilitates charge transport and suppresses charge recombination. As a result, WD-6-based binary OPVs using PM6 as the donor achieve a power conversion efficiency (PCE) of 17.44%, which was further improved to 18.41% with 2PACz as the hole transport layer. Then, with WD-6 as a guest acceptor, the PM6:BTP-eC9:WD-6 ternary device achieves an impressive PCE of 19.42%. Besides, the WD-6-based binary OPD also achieves a response time of 205 ns when operating in self-powered mode. Notably, this marks the highest efficiency (Table S1) and fastest response speed achieved so far in binary optoelectronics based on rigid multi-arm small-molecule acceptors. These findings provide new perspectives for designing organic semiconductor materials simultaneously exhibiting high photovoltaic and photodetective performance.

## RESULTS

The synthetic route of WD-6 is illustrated in Scheme S1, and the detailed procedures for synthesis and characterizations are provided in the supplementary data. The molecule exhibits good solubility in common solvents such as chloroform and chlorobenzene, and shows high thermal stability, with a decomposition temperature (T_d_) of 318°C from the thermal gravimetric analyzer (TGA) analysis (Fig. S1). The surface electrostatic potential (ESP) distribution and dipole moment of the model molecules was analyzed. As shown in Fig. 1b, most of the surfaces of the molecule show positive ESP, indicating its electron affinity characteristics. And compared to Y6, with a small dipole moment (μ) of 0.87 Debye [26], WD-6 exhibits a large μ of 3.22 Debye, indicating that the molecular skeleton is not planar, confirmed by the single crystal analysis discussed below. The large dipole moment is beneficial for the formation of the intense intermolecular packing [27]. On the molecular level, reorganization energy is used to characterize the deformations of the molecular structure during the electron-transfer process [28]. According to Marcus charge-transfer theory, a small reorganization is conducive to reducing the driving force required for exciton dissociation and improving charge mobility [29]. Notably, WD-6 shows a very low computed electron reorganization and hole reorganization energy with values of 96.38mev and 108.44mev, respectively (Fig. S2), which are lower than that of most Y6 derivatives reported to date [30,31]. The small reorganization energy of WD-6 is further confirmed by the narrow Stokes shift of WD-6 in film. As displayed in Fig. 1c, the Stokes shift value of WD-6 is only 52 nm, also smaller than that of most Y6 derivatives [28,32,33].

The *E*_U_, an important parameter to characterize the energetic disorder, was quantificationally investigated in the WD-6 thin film using Fourier transform photocurrent spectroscopy-external quantum efficiency (FTPS-EQE) [34,35]. The calculation formula of *E*_U_ is:


(1) \begin{eqnarray*}E_{\rm U}\!\left( E \right) = {{\left[ {\frac{{{\rm dln}\!\left( {EQE} \right)}}{{{\rm d}E}}} \right]}^{ - 1}},\end{eqnarray*}

where E is the photon energy and EQE is the external quantum efficiency. We fabricated a single-component device with the structure of indium tin oxide (ITO)/poly(3,4-ethylenedioxythiophene)-poly(styrenesulfonate) (PEDOT:PSS)/WD-6/PNDIT-F3N/Ag. The *E*_U_ is calculated to be 21.7 meV through the exponential fitting of the sub-bandgap FTPS-EQE spectrum (Fig. 1d). To our knowledge, the value of 21.7 meV is among the lowest *E*_U_ reported for organic semiconductor materials [8,36]. The low *E*_U_ of WD-6 can be attributed to the molecular rigidity and intermolecular interaction of WD-6 owing to its 2D conjugate extension and multi-arm structure. These characteristics favor the promotion of carrier transport, thereby improving the performance of organic semiconductors.

The intermolecular packing morphology and crystallinity of the WD-6 neat film were studied using grazing incidence wide-angle X-ray scattering (GIWAXS) (Fig. 1e). As plotted line-cut profiles in Fig. 1f and summarized parameters in Table S2 show, the WD-6 neat film exhibits strong π−π stacking (010) diffraction peaks in the out-of-plane (OOP) direction. The π−π distance and π−π stacking crystalline coherence length (CCL) of WD-6 are calculated to be 3.88 Å and 18.18 Å, respectively, indicating that WD-6 possesses ordered molecular packing and good crystallinity, which contributes to its low *E*_U_ [36–38], small reorganization energy and high electron mobility of 3.39 × 10^–4^ cm^2^ V^–1^ s^–1^ (Figs S3 and S4, average electron mobility is 3.38 × 10^−4^ cm^2^ V^−1^ s^−1^).

**Figure 2.**
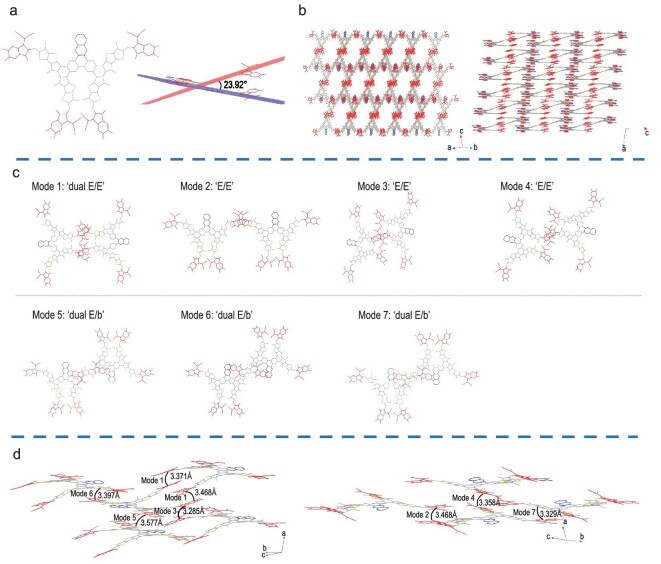
(a) The top view and side view for the single crystal structure of WD-6. (b) Single-crystal 3D-packing topological structures. (c) Intermolecular packing modes. E and b represent end and bridge units, respectively. (d) π–π stacking distances of the interlayer including all the main molecular packing modes.

The single crystal of WD-6 was obtained through solve diffusion. As shown in Fig. 2a, the top view indicates that the backbone of WD-6 is constituted by two wings, giving it the appearance of a butterfly. These two wings are not coplanar with each other, as indicated by the side view showing a twist of 23.92°. This twist should be caused by the large steric hindrance between the long side chains on the thiophene units. The butterfly-shaped and rigid skeleton structure can benefit the formation of strong intermolecular interaction. Figure 2b shows that a distinctive 3D packing network is well formed, facilitating charge transport in the corresponding optoelectronic devices. Notably, the particularity of the WD-6 butterfly-shaped skeleton structure gives rise to different intermolecular packing modes. As illustrated in Fig. 2c and d, and Fig. S5, seven main packing modes can be observed for WD-6, including a dual end-to-end mode (dual E/E), three types of end-to-end modes (E/E) and three types of dual end-to-bridge modes (dual E/b). Among them, ‘dual E/E (mode 1)’ for WD-6 is a distinctive mode newly observed in organic photoelectric materials, in which each WD-6 molecule undergoes efficient π–π stacking with the adjacent molecules through the two terminal groups with a π–π stacking distance of 3.371 Å. In short, a unique and condensed 3D packing network is formed in the crystal of WD-6 primarily owing to its butterfly-shaped and rigid skeleton structure, which explains why it shows low energy disorder and small reorganization energy.

**Figure 3.**
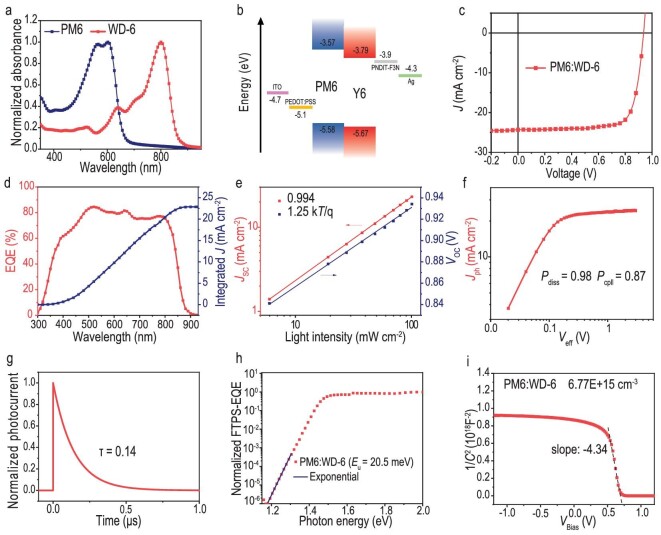
(a) Absorption spectra of PM6 and WD-6 in solid films. (b) The alignment of energy levels. (c) *J*–*V* curves for OPVs. (d) EQE plots and integrated *J*_SC_ curves. (e) Light intensity (*P*_light_) dependence of *J*_SC_ and *V*_OC_. (f) *J*_ph_ versus *V*_eff_ curves indicating *P*_diss_ and *P*_coll_. (g) Transient photocurrent measurement of the PM6:WD-6-based device. (h) FTPS-EQE of the PM6:WD-6 device at the absorption onset. (i) Mott–Schottky plots (dashed lines represent the linear fitting).

As illustrated in Fig. 3a, the maximum absorption peak of the WD-6 neat film is located at 800 nm. It shows a redshift with 73 nm from its solution to film, suggesting strong intermolecular π–π interactions (Fig. S6). The energy levels of WD-6 were estimated by cyclic voltammetry (CV) (Fig. S7). From the CV curves, the HOMO level and the LUMO energy levels are determined to be −5.67 and −3.79 eV, respectively (Fig. 3b). Considering the complementary absorptions and matched energy levels, PM6 was selected as the donor polymer material to study the photovoltaic performance of WD-6. OPVs with the structure of ITO/PEDOT:PSS/PM6:WD-6/PNDIT-F3N/Ag were fabricated. The detailed device fabrication process is provided in the supplementary data and the structures of PM6, PEDOT: PSS and PNDIT-F3N are shown in Figs S8 and S9. After optimization, WD-6-based OPV devices exhibited an excellent PCE of 17.44%, with a short-circuit current density (*J_SC_*) of 24.29 mA cm^−2^, an open-circuit voltage (*V_OC_*) of 0.937 V and a fill factor (FF) of 76.61% (Fig. 3c). The EQE curve of the optimized device is depicted in Fig. 3d. The integrated current density from the EQE curve is 23.20 mA/cm^−2^, matching well with that measured in the current density-voltage (*J-V*) test.

The exciton and charge dynamic analysis was conducted to investigate the PM6:WD-6-based OPV device. Firstly, charge mobilities of the blend film were measured by the space charge limited current (SCLC) method. As depicted in Fig. S10, the blend film exhibited the hole mobility and electron mobility to be 4.59 × 10^−4^ and 3.88 × 10^−4^ cm^2^ V^−1^ s^−1^, respectively. The average hole mobilities (4.53 × 10^−4^ cm^2^ V^−1^ s^−1^) and electron mobilities (3.65 × 10^−4^ cm^2^ V^−1^ s^−1^) of the blended film were also provided for fair comparison, respectively (Fig. S4). The high and balanced hole/electron mobility ratio for PM6:WD-6 blend film favored the high FF and *J*_SC_ in the photovoltaic device. Next, the charge recombination under the short-circuit condition was quantified by the power-law dependence of *J*_SC_ on light intensity. The equation is *J_SC_* ∝ *P*_light_^α^. If α approaches 1, bimolecular recombination is almost negligible. The difference of recombination mechanisms in the open-circuit condition can be characterized by the dependence of *V_OC_* and the natural logarithm of light intensity. The slope is nkT/q (k is Boltzmann constant, T is thermodynamic temperature and q is elementary electronic charge); the closer n is to 1, the less trap-assisted recombination [39]. As shown in Fig. 3e, the result indicates less bimolecular recombination (α = 0.994) and trap-assisted recombination (*n* = 1.25) in the device. Benefitting from high carrier mobilities and less carrier recombination, the OPV device showed a high exciton dissociation probability (*P*_diss_ = 0.98) and charge extraction efficiency (*P*_coll_ = 0.87), as shown by the photogenerated current density (*J*_ph_) versus effective voltage (*V*_eff_) curves [40] (Fig. 3f).

The transient photocurrent (TPC) test was used to further explore the carrier dynamics. As plotted in Fig. 3g, the charge extraction time is only 0.14 μs, which is also one of the reasons for the high PCE of the WD-6-based device. Furthermore, the *E*_U_ of PM6:WD-6 blend film was obtained by exponentially fitting its sub-bandgap FTPS-EQE spectrum (Fig. 3h). The blend film has an impressively low *E*_U_ of 20.5 meV, which is among the lowest *E*_U_ values ever reported for the OPV device [30,36,41]. The decreased *E*_U_ in the blend film may be attributed to the improved thin-film crystallinity and tightness after introducing the highly crystalline polymer PM6 into the amorphous organic system [8]. Mott–Schottky analysis at various bias voltages (Fig. 3i) showed that the PM6:WD-6 film had a low trap density of 6.77 × 10^15^ cm^−3^. The low *E*_U_ and trap density imply less disorder in the blend film. Then, considering the impact of energy disorder on voltage loss, a detailed energy loss (E_loss_) analysis was conducted (Fig. S11, Table S3). PM6:WD-6-based OPV has a small ∆E_2_ (radiative recombination loss below the bandgap) of 0.040 eV, which agrees with the small reorganization energy of WD-6 and low energetic disorder of PM6:WD-6. Moreover, the device showed a low non-radiative energy loss (∆E_3_) with a value of 0.215 eV.

**Figure 4.**
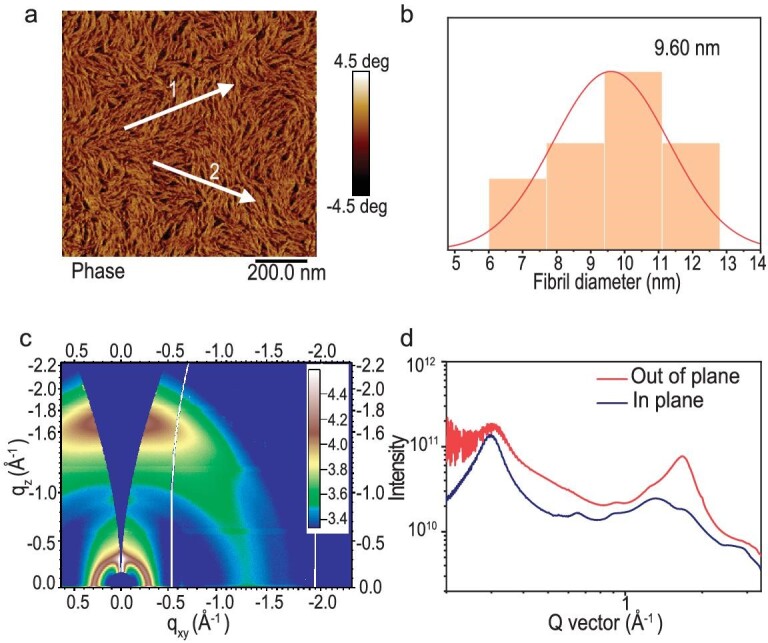
(a) AFM phase images of blended film. (b) Statistical distribution of fibril diameters. (c) 2D GIWAXS patterns of blended film. (d) Extracted line-cut profiles from 2D GIWAXS patterns of blended film.

To investigate the correlation between the morphology and device performance, atomic force microscopy (AFM) and GIWAXS were conducted on the blending film of PM6:WD-6. As illustrated in Fig. 4a and b, and Fig. S12, the blend film has a distinct fibrillar network interpenetrating structure with a statistic diameter of 9.6 nm. In addition, the blend film also has relatively smooth surfaces with a surface roughness of 1.24 nm. Note that the smooth surfaces, and these nanofiber structures, are expected to decrease charge recombination and promote charge separation and transport. From the GIWAXS analysis of the blend film of PM6:WD-6 (Fig. 4c and d; Table S4), the film has a strong π–π stacking peak in the OOP direction at 1.65 Å^−1^ (d = 3.8 Å) and a strong lamellar peak in the in-plane (IP) direction at 0.296 Å^−1^ (d = 21.19 Å), which indicates that the blend film adopts a preferential face-on stacking pattern. Furthermore, in the IP direction, the CCL for the lamellar stacking of the blend film is 87.00 Å, which is larger than that of neat film (49.60 Å). Thus, blend film exhibits less disorder and higher crystallinity which contributes to the low *E*_U_ in the blend film.

**Figure 5.**
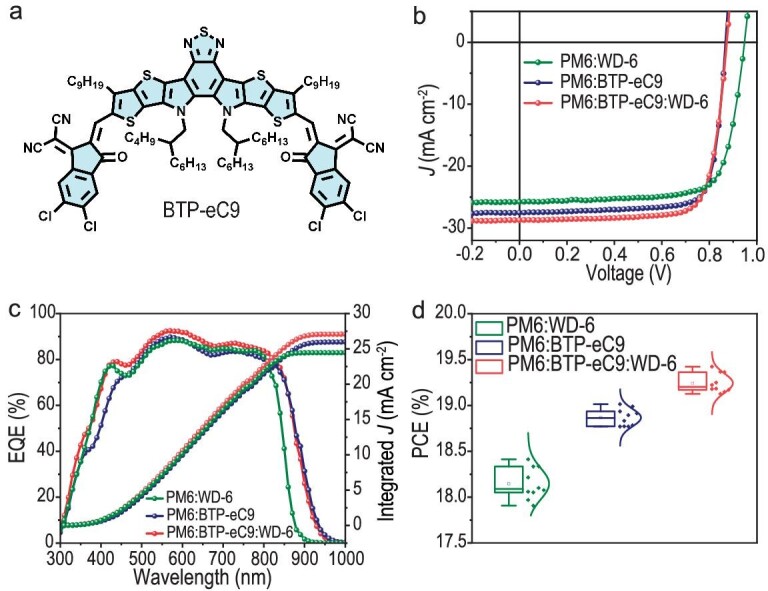
(a) Chemical structures of BTP-eC9. (b) *J-V* characteristics of OPVs based on PM6:WD-6, PM6:BTP-eC9 and PM6:BTP-eC9:WD-6. (c) EQE spectra and the integrated *J*_SC_ of OPVs based on PM6:WD-6, PM6:BTP-eC9 and PM6:BTP-eC9:WD-6. (d) Statistical histograms of PCEs of OPVs based on PM6:WD-6, PM6:BTP-eC9 and PM6:BTP-eC9:WD-6.

Considering that 2PACz (Fig. S9) as a hole transport layer (HTL) can mitigate parasitic absorption loss and induce lower contact resistance [42,43], we employed 2PACz to replace PEDOT:PSS in fabricating binary devices in order to achieve higher device efficiency. As a result, the PCE of the devices based on PM6:WD-6 could be further improved to 18.41%, marking the highest PCE reported for rigid multi-arm small molecule acceptors. In addition to interface engineering strategies, efforts focused on the ternary blend active layer can significantly contribute to enhancing device performance [44,45]. Here, we fabricated ternary devices using BTP-eC9 as the second acceptor as it has broader absorption than WD-6. The chemical structure of BTP-eC9 is illustrated in Fig. 5a. The device structure of the ternary solar cell is ITO/2PACz/PM6:BTP-eC9:WD-6 (1 : 1 : 0.2)/PNDIT-F3N/Ag. After device optimization, a remarkable PCE of 19.42%, with a *V*_oc_ of 0.873 V, a *J*_sc_ of 28.72 mA cm^−2^ and an FF of 77.47% was achieved for the ternary device. The *J-V* curves for the two binary and the ternary devices are provided in Fig. 5b and the photovoltaic parameters are listed in Table 1. Figure 5c shows the EQE curves of the binary and ternary devices, from which the integrated current densities were calculated. These current densities are consistent with the *J*_SC_ values from the *J*-*V* test. Moreover, these devices exhibited good reliability, as shown in Fig. 5d, a conclusion arrived at by measuring 10 independent devices.

**Table 1.** Summary of photovoltaic parameters for OPVs.

Active layers	*V*_OC_ (V)	*J*_SC_ (mA/cm^2^)	*J*_SC_^EQE^ (mA/cm^2^)	FF (%)	PCE (%)^a^
PM6:WD-6	0.948 (0.946 ± 0.003)	25.77 (25.73 ± 0.15)	24.84	75.37 (74.56 ± 0.50)	18.41 (18.15 ± 0.17)
PM6:BTP-eC9	0.869 (0.857 ± 0.007)	27.52 (27.98 ± 0.29)	26.34	79.51 (78.64 ± 0.78)	19.01 (18.87 ± 0.09)
PM6:BTP-eC9:WD-6(20%)	0.873 (0.873 ± 0.003)	28.72 (28.37 ± 0.30)	27.47	77.47 (77.69 ± 0.47)	19.42 (19.24 ± 0.10)

a The average values in parentheses were obtained from 10 independent devices.

According to the Shockley-Read-Hall (SRH) theory, in the dark, electrons are thermally excited from the edge of the donor HOMO (charge-transfer (CT) ground state) to the acceptor LUMO with the assistance of trap states, which contributes to the noise current in OPDs [11,46]. Additionally, charge transport in highly disordered amorphous organic films generally occurs via the hopping process, requiring sufficient energy to overcome the barriers caused by energetic disorders, as described by the Arrhenius-like law [47]:


(2) \begin{eqnarray*}{{\mu }_0} = {{\mu }_\infty }\exp \left( { - \frac{\Delta }{{{{k}_{\mathrm{B}}}T}}} \right).
\end{eqnarray*}

Here, Δ represents the activation energy, which increases with the number of disorders. Assuming that the traps are homogeneously dispersed, the mobility can be expressed as:


(3) \begin{eqnarray*}\mu = {{\mu }_0}\alpha \exp \left( { - \frac{{{{E}_{\mathrm{t}}}}}{{kT}}} \right),\end{eqnarray*}

where *E*_t_ is the trapping energy and $\alpha $ is the ratio of the density of delocalized levels available for transport to the density of traps. Therefore, reducing trap states and energetic disorder in photoactive films can effectively suppress the noise current and enhance charge mobilities, thus leading to high sensitivity and response speed in OPDs.

**Figure 6.**
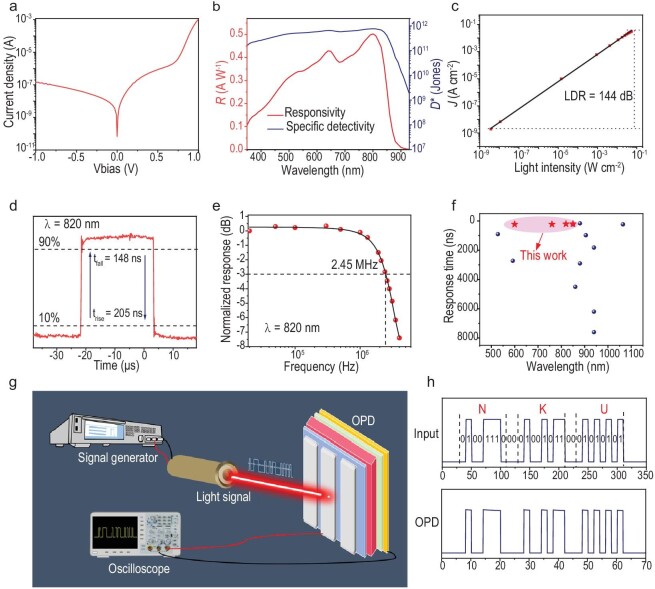
(a) *J*–*V* curves in the dark. (b) Responsivities and specific detectivities of the OPD working at 0 V bias voltage. (c) LDR of the PM6:WD-6-based OPD under the irradiation of NIR light (λ=820 nm) at zero bias. (d) Time domain response of PM6:WD-6-based OPD. (e) −3 dB cutoff frequency under 820 nm illumination. (f) Summarization of high-speed OPDs, with references 1–11 relating to high-speed OPDs operating without external bias (Table S6). (g) Schematic of the optical communication system. (h) The input information and output signals under NIR light (820 nm).

Benefitting from the low trap density and minimal energetic disorder in the PM6:WD-6 blend film, the WD-6 has great potential in OPDs with a high sensitivity and fast response. To validate this assumption, we fabricated OPDs with an inverted device structure of ITO/ZnO/NMA/PM6:WD-6/MoO3/Ag. The detailed device fabrication process is presented in the supplementary data. To suppress the noise current and improve the sensitivity of the OPDs, we conducted all the measurements of the OPDs in the photovoltaic mode. As displayed in Fig. 6a, the OPD with WD-6 shows a low dark current of 6.8 × 10^−11^A/cm^2^ in the self-powered mode, ensuring a low shot noise. Responsivity (*R*) is a crucial parameter for assessing the sensitivity of an OPD, which is calculated via the following equation [48]:


(4) \begin{eqnarray*}R = \frac{{EQE}}{{100\% }} \times \frac{{{{\lambda }_{\textit{input}}}}}{{1240\left( {{\rm nm}\ {\rm W}\ {{\rm A}^{ - 1}}} \right)}},\end{eqnarray*}

where ${{\lambda }_{\textit{input}}}$ is the incident light wavelength. As shown in Fig. 6b, the WD-6-based OPD exhibited a high responsivity from the UV region (300 nm) to the near-infrared (NIR) region (878 nm) under 0 V, peaking at 0.50 A W^−1^ in 800 nm, which is close to the commercial high-speed Si photodetectors (PDs) within the same spectrum [5] (Model S1133-01).

The specific detectivity (D*) is another key figure of merit to evaluate the sensitivity of OPDs for detecting ultraweak incident light signals. Combining the responsivity and noise current, the D* can be extracted using the following equation [48]:


(5) \begin{eqnarray*}{{D}^*} = \frac{{R\sqrt {AB} }}{{{{i}_n}}},\end{eqnarray*}

where *A* is the effective device area, *B* is the bandwidth and *i_n_* is the noise current. The noise current in the OPD, encompassing shot noise (*i_shot_*), thermal noise (*i_thermal_*) and frequency-dependent flicker noise (*i_1/f_*), plays a substantial role in the D*. The total noise current (${{i}_n}$) can be calculated as follows [49]:


(6) \begin{eqnarray*}{{i}_n} = \sqrt {i_{\textit{shot}}^2 + i_{\textit{thermal}}^2 + i_{\frac{1}{f}}^2}, \end{eqnarray*}

(7) \begin{eqnarray*}{{i}_{\textit{shot}}} = \sqrt {2e{{I}_{\textit{dark}}}B}, \end{eqnarray*}

(8) \begin{eqnarray*}{{i}_{\textit{thermal}}} = \sqrt {\frac{{4{{k}_B}TB}}{{{{R}_{sh}}}}}, \end{eqnarray*}

where *e* represents the elementary charge, *I*_d_ is the dark current, *k*_B_ is the Boltzmann constant, *T* is the absolute temperature and *R*_sh_ is the shunt resistance of the device. To accurately measure the total current noise (*i_n_*), it is common to measure the dark current of OPD, followed by detracting the *i_n_* using Fourier Transform approach , with the D* subsequently calculated using Equation (5). As shown in Fig. S14, the measured *i_n_* reached 1.31 × 10^−13^ A Hz ^−1/2^ in self-powered mode, along with an average D* of 10^11^ *Jones* across a range of 300–870 nm, with a peak value of 7.7 × 10^11^ *Jones* at 800 nm (Fig. 6b).

The linear dynamic range (LDR) is commonly utilized to assess the adaptation ability of OPDs under a series of light intensities from highly dark to bright environments, which heavily depends on the responsivity and noise current of OPDs. The LDR is extracted from the following equation [48]:


(9) \begin{eqnarray*}LDR = 20\log \left( {\frac{{{{J}_{\rm max}}}}{{{{J}_{\rm min}}}}} \right).
\end{eqnarray*}

As shown in Fig. 6c, the WD-6-based OPD exhibits a linear response covering nine orders of magnitude under a light irradiation of 820 nm, which is over 144 dB due to the high responsivity and low noise current.

In the wireless optical communication system, the response speed of OPD plays the most crucial role in receiving high-capacity and high-speed data. Here, we measured the response/recover time of the OPD via the steady-state analysis technique instead of transient photocurrent measurement. As shown in Fig. 6d, under a pulsatile illumination of 820 nm, the response/recover times of the OPD-based WD-6 achieve 205 and 148 ns at zero bias, respectively, which are some of the fastest responses among binary OPDs processed using the solution technique. Moreover, the OPD exhibits an impressive response speed across the whole spectrum, from visible to NIR regions (Fig. S15). It is worth noting that the response/recover speed of WD-6-based OPDs even surpasses most of the popular commercial Si PDs, demonstrating great potential in high-speed wireless optical communication [50]. Theoretically, the data transit volume and speed are primarily determined by the OPD's −3 dB cutoff frequency (f-3 dB). As shown in Fig. 6e, the f-3 dB of an OPD with WD-6 achieves 2.45 MHz, which is one of the superior data of OPDs reported recently. As summarized in Fig. 6f, the OPD based on PM6:WD-6 has the highest response speed among the reported devices in the 600–820 nm wavelength range. All these superior performances are attributed to the low energetic disorder and low trap states in the photoactive films based on PM6:WD-6.

To verify the feasibility of the high-speed Vis-NIR OPD in practical wireless optical communication, we integrated the OPD as an optical signal receiver into the optical communication system for data transmission. Figure 6g displays the schematic of the optical communication system, which combines the signal generator, LED, OPD and oscilloscope. By programming information using the signal generation that converts data between high and low voltage levels, driving the LED to emit lights with different intensity, the OPD receives the optical signal and converts it into electric signals, which are then collected and displayed on the oscilloscope. Figure 6h presents the input information and output signals under NIR light (820 nm), showing a high level of consistency. Leveraging the high-speed response of our OPD, we designed and assembled a text communication system by combining modern radio communication technology with the optical communication system, achieving high-speed wireless optical communication via NIR light (Supplementary  Video). By sending text information from a mobile phone to the microcontroller that can modulate the NIR LED, the LED will emit an optical signal that the NIR OPD collects and converts into electric signals.

## DISCUSSION

In summary, we designed and synthesized a new butterfly-shaped dimer with a 2D conjugation extension and rigid core. The molecule exhibits a low *E*_U_ and small reorganization energy due to its rigid and large conjugated backbone, ordered molecular packing and good crystallinity. A WD-6-based binary photovoltaic device achieved an efficiency of 18.41%, one of the highest values among binary cells based on rigid multi-arm small-molecule acceptors. Impressively, a PCE of 19.42% was achieved for the ternary device based on PM6:BTP-eC9:WD-6. Furthermore, the WD-6-based OPD demonstrated a fast response/recover speed across the spectrum, from 600 to 850 nm, which even surpasses the popular commercial Si PD. We have also showcased the potential practical application of WD-6-based OPDs with a rapid response and a high cutoff frequency of −3 dB in next-generation optical communication technology. Overall, our result demonstrates a new molecular design strategy involving rigid and large conjugated acceptors, and provides valuable molecular design guidelines for developing efficient OPVs and high-speed OPDs.

## MATERIALS AND METHODS

Detailed materials and methods are available in the online supplementary data.

## Supplementary Material

nwae409_Supplemental_File
