# Supplementary material for: A butterfly-shaped acceptor with rigid skeleton and unique assembly enables both efficient organic photovoltaics and high-speed organic photodetectors
Source: Natl Sci Rev. 2024 Nov 13;12(1):nwae409. doi: 10.1093/nsr/nwae409 (PMC11702656; doi:10.1093/nsr/nwae409)
Supplement: nwae409_Supplemental_File [file nwae409_supplemental_file.zip › Teaser text.docx]

A butterfly-shaped bifunctional acceptor named WD-6 has been designed, which achieves a high efficiency of 19.42% in OPV and an ultra-fast response speed of 205 ns in OPD.
